# Design of a factorial experiment with randomization restrictions to assess medical device performance on vascular tissue

**DOI:** 10.1186/1471-2288-11-75

**Published:** 2011-05-20

**Authors:** Wiebke S Diestelkamp, Carissa M Krane, Margaret F Pinnell

**Affiliations:** 1 Department of Mathematics, University of Dayton, 300 College Park Ave., Dayton, OH 45469, USA; 2 Department of Biology, University of Dayton, 300 College Park Ave., Dayton, OH 45469, USA; 3 Department of Mechanical and Aerospace Engineering, University of Dayton, 300 College Park Ave., Dayton, OH 45469, USA

**Keywords:** Factorial design, split-plot design, randomization restrictions, vascular, biostatistics, burst pressure

## Abstract

**Background:**

Energy-based surgical scalpels are designed to efficiently transect and seal blood vessels using thermal energy to promote protein denaturation and coagulation. Assessment and design improvement of ultrasonic scalpel performance relies on both in vivo and ex vivo testing. The objective of this work was to design and implement a robust, experimental test matrix with randomization restrictions and predictive statistical power, which allowed for identification of those experimental variables that may affect the quality of the seal obtained ex vivo.

**Methods:**

The design of the experiment included three factors: temperature (two levels); the type of solution used to perfuse the artery during transection (three types); and artery type (two types) resulting in a total of twelve possible treatment combinations. Burst pressures of porcine carotid and renal arteries sealed ex vivo were assigned as the response variable.

**Results:**

The experimental test matrix was designed and carried out as a split-plot experiment in order to assess the contributions of several variables and their interactions while accounting for randomization restrictions present in the experimental setup. The statistical software package SAS was utilized and PROC MIXED was used to account for the randomization restrictions in the split-plot design. The combination of temperature, solution, and vessel type had a statistically significant impact on seal quality.

**Conclusions:**

The design and implementation of a split-plot experimental test-matrix provided a mechanism for addressing the existing technical randomization restrictions of ex vivo ultrasonic scalpel performance testing, while preserving the ability to examine the potential effects of independent factors or variables. This method for generating the experimental design and the statistical analyses of the resulting data are adaptable to a wide variety of experimental problems involving large-scale tissue-based studies of medical or experimental device efficacy and performance.

## Background

Blood vessel transection and sealing requires that tissue segments be held together with an immediate and temporary bond that prevents leakage and holds long enough to allow the adjacent tissue margins to re-grow, heal, and regenerate structural connections through the natural healing process. Energy-based vessel sealing methods use no exogenous materials to accomplish vessel closure, but rely on acute vessel compression, thermally induced tissue injury, and tissue healing to generate the seal. This process limits inflammation and eliminates concerns with biocompatibility and immunogenicity associated with using an exogenous material such as sutures, staples, or bonding adhesives. In addition, energy-based methods are easier and faster to use than materials-based methods, especially for endoscopic surgeries, resulting in a decrease in operative time and surgical invasiveness, and reduced infection rates. Ultrasonic scalpel technology has been used extensively in fine tissue dissection, hemostasis, vessel closure and transection [1-3]. The optimization of ultrasonic device performance relies on both in vivo animal testing and ex vivo tissue testing at a level and degree that achieves statistically significant evidence of performance standards. Many physiological and anatomical properties of biological tissues exhibit inter-individual variability due to the sex, age, and health of the animal from which the tissue was harvested. Moreover, it is technically difficult to recapitulate physiological conditions ex vivo. However, proof of device efficacy through ex vivo testing is required prior to in vivo testing. As such, it is critical to control as many experimental variables as possible, and to measure those that cannot be controlled during ex vivo testing in order to achieve results that realistically assess device performance. Therefore, the challenge is to identify those experimental conditions that should be tested, and those that should be controlled. The generation of statistically relevant observations is dependent on the careful design of a test matrix that facilitates the assessment of the effects of fixed and variable test conditions while taking into account technical or procedural restrictions to randomization that exist for the proposed experiments. In this report, the computational and biostatistical tools that were employed to generate a robust and randomized test matrix to assess ex vivo ultrasonic scalpel performance are described.

## Methods

### Porcine vessel sealing and burst pressure test

Porcine vessel sealing and burst pressure testing were performed as previously described [4]. Briefly, porcine carotid and renal arteries were harvested from tissue remnants from animals slaughtered for food consumption at a USDA-inspected slaughterhouse facility by technicians from Animal Technologies (Tylersville, TX). Institutional Animal Care and Use Committee (IACUC) approval was not required for the use of these tissue remnants since no live animals were used in experiments, and the tissues were obtained from a food-purposed slaughterhouse source. Luer locks were sutured to each end of the artery. The arteries were mounted onto a custom-designed tension device that stretched the vessel at a constant rate of 0.7 mm/s to a load of ~113 gram-force (0.25 lbf). The tensioned vessel was infused with one of three test solutions pre-equilibrated to ambient temperature (23°C) or 37°C. An HA2001 PHD2000 Harvard Apparatus dual syringe infusion pump supplied a constant infusion rate of 1.5 ml/min. During infusion, each artery was transected and sealed using an ultrasonic scalpel connected to an ultrasonic generator set at power level 3. Subsequent to transection and sealing, each vessel was subjected to pressure burst testing. A 10,340 mmHg (200 psi) Measurements Specialties MSP-300-200-P-N-1- pressure transducer was used to record the pressure that was generated in the sealed vessel during constant infusion with the test solution at a rate of 20 ml/min. Vessel burst pressure, the response variable in this experiment, was defined as the pressure at which the test solution was observed to leak from the seal and/or a rapid drop in pressure was noted in the pressure versus time curve [5,6]. The load cell and pressure transducers were connected to a Vishay Micromeasurements System 5000 Model 5100 B Scanner and Strain Smart^® ^software was used to record the data.

### Biostatistical experimental matrix design

The design included three factors: The temperature of the solution (Temp - two levels; ambient (23°C) or 37°C), the type of solution that was utilized (Sol - three types; 0.9% saline; 6% Hetastarch (HES); "Plasma protein" enriched buffer (95% Hanks balanced salt solution, 4.7% bovine serum albumin fraction V, 0.36% fibrinogen type IV)) and the type of vascular tissue used (Vessel - two types; carotid or renal artery).

### Sample size calculation

A number of preliminary experiments (n = 4-12) were carried out as described above. The data were used to perform basic sample size computations to aid in determining the sample size for the main experiment. All computations were based on comparisons of two means. To determine the sample size necessary to detect a significant difference between the means of two factors at a desired significance level and with given power, the observed sample means and the pooled standard deviation corresponding to the two factors from the pilot were computed. The Analyst application in SAS was utilized to compute the sample sizes. The input consisted of the two observed sample means, the pooled standard deviation, the desired significance level and the desired power for the test.

### Statistical analyses of the data

The statistical package SAS was used to carry out the data analysis. The factors temperature, solution and vessel type, as well as "Session" (with 48 levels) were declared as class variables for the analysis of variance. To incorporate the information about the randomization restrictions discussed above, PROC MIXED was used, and Session was declared as a random effect as suggested in [7] (the others are treated as fixed effects by default). The data set was imported into SAS from Excel, with columns for session, temperature, solution, vessel type and the response variable.

### SAS code

PROC MIXED data = dataset;

CLASS Session Temp Sol Vessel;

MODEL response = Temp|Sol|Vessel;

RANDOM session;

RUN;

### Interpretation of SAS output

SAS computed the ANOVA table displaying the p-values for the main effects (temperature, solution and vessel type) and their interactions. Using the significance level of 0.05, a p-value of less than 0.05 indicates a (statistically) significant main effect or interaction effect.

## Results

The objective of this work was to design and implement a robust, experimental test matrix with randomization restrictions and predictive statistical power, which allowed for identification of those experimental variables that may affect the quality of the seal obtained ex vivo. A number of pilot studies using small sample sizes were initially performed to determine which variables likely contribute to seal quality and therefore should be included in the test design. The factors initially studied included temperature of the solution, type of infusion solution, vessel type, and infusion flow rate during blood vessel transection/sealing. The response variable in each case was the burst pressure (mmHg) at which the seal failed.

In addition, the pilot data was used to estimate the sample size needed to obtain significant results (at a significance level of 0.05) with a power of 0.9, or the probability that a test with the specified assumptions (sample size, observed difference of means, observed pooled standard deviation, and type of alternative hypothesis) would correctly reject the null hypothesis when the alternative hypothesis was true. Given the minimum desired detectable difference, as well as (an estimation for) the standard deviation and the desired significance level of the test, the sample size that was necessary for a two-sample test to have a given power was computed. The sample size computations were based on pairwise comparisons, in which each factor level was examined as to its potential effect on burst pressure. In some cases, it was clear that these comparisons were not trending towards showing significant differences, and the corresponding factors (or factor levels) were therefore not included in the experimental design. However, those comparisons that were not statistically significant in the pilot study due to small sample size but showed a trend toward predicted statistical significant differences with an increased sample size (based on the power calculation) were included in the experimental design. In addition, the interaction effects of the included factors (comparisons between combinations of two or three factors) were deemed important to assess.

Based on the pilot studies, the effects of solution temperature, solution type, and vessel type were chosen to be studied. Taking into account the sample size computations made from the pilot study and the practical limitations imposed by the experimental test setup, the size of the matrix and experimental design was determined. A balanced design for the test matrix was selected where each treatment combination was observed the same number of times. The experiment included three factors, with a total of 12 possible treatment combinations. While the vessel type could be varied at random from run to run, the temperature and solution were hard-to-change factors. This physical reality forced randomization restrictions on the design. The experiment consisted of 480 runs (40 per treatment combination), divided into 48 sessions of 10 runs each. For each session, one of the six combinations of levels for temperature and solution was chosen (each combination was used for eight different sessions) and held fixed. The assignment of the treatment combinations to the sessions was carried out at random. This assignment was done in Excel. Using a column for temperature and a column for solution, eight copies of the treatment combinations of temperature and solution were generated. A third column contained random numbers. The data in the three columns was then sorted in ascending order of the random numbers to create a random order of the treatment combinations. The treatment combinations were then assigned to the 48 sessions in that order. Each session contained ten runs (five runs per vessel type) chosen in random order using the same procedure in Excel. The procedure to generate a random order of the sessions is shown in Table 1. For simplicity, the procedure is demonstrated for the case of only 12 sessions, where each Temperature/Solution combination is used for two sessions. The random numbers in the third column were generated using the rand() function (which generates a random number between 0 and 1). Next, the three columns were sorted by the size of the random numbers (Table 2). This resulted in a random ordering of the 12 rows (sessions; Table 3). For example, in session 1, Temperature 1 and Solution 3 were used throughout, while in session 2, Temperature 2 and Solution 2 were used for all runs.

**Table 1:** Two copies of the six Temperature/Solution combinations, plus a column of random numbers.

Temperature	Solution	Random number
Temp1	Sol1	0.617940611

Temp1	Sol2	0.739488341

Temp1	Sol3	0.088714721

Temp2	Sol1	0.556090887

Temp2	Sol2	0.55227804

Temp2	Sol3	0.691030612

Temp1	Sol1	0.286876071

Temp1	Sol2	0.535759581

Temp1	Sol3	0.882488882

Temp2	Sol1	0.981425728

Temp2	Sol2	0.228304389

Temp2	Sol3	0.606414609

**Table 2:** Two copies of the six Temperature/Solution combinations, ordered by the size of the random numbers.

Temperature	Solution	Random number
Temp1	Sol3	0.088714721

Temp2	Sol2	0.228304389

Temp1	Sol1	0.286876071

Temp1	Sol2	0.535759581

Temp2	Sol2	0.55227804

Temp2	Sol1	0.556090887

Temp2	Sol3	0.606414609

Temp1	Sol1	0.617940611

Temp2	Sol3	0.691030612

Temp1	Sol2	0.739488341

Temp1	Sol3	0.882488882

Temp2	Sol1	0.981425728

**Table 3:** The twelve sessions in random order.

Session	Temperature	Solution
1	Temp1	Sol3

2	Temp2	Sol2

3	Temp1	Sol1

4	Temp1	Sol2

5	Temp2	Sol2

6	Temp2	Sol1

7	Temp2	Sol3

8	Temp1	Sol1

9	Temp2	Sol3

10	Temp1	Sol2

11	Temp1	Sol3

12	Temp2	Sol1

An analysis of variance was conducted to determine the effects of the factors and their interactions. The resulting ANOVA table is shown in Table 4. For this data set, there were no significant main effects due to vessel type, solution temperature, or type of solution, nor were there significant two-factor interactions (at the 0.05 significance level). Only the 3-factor interaction between temperature, solution and vessel type was significant at 0.05, with a p-value of 0.027.

**Table 4:** ANOVA Table.

Effect	Num DF	Den DF	F Value	Pr > F
Sol	2	426	0.21	0.812

Temp	1	426	1.14	0.2857

Sol*Temp	2	426	0.05	0.949

Vessel	1	426	2.65	0.1045

Sol*Vessel	2	426	0.83	0.4354

Temp*Vessel	1	426	1.36	0.2449

Sol*Temp*Vessel	2	426	3.64	0.027

Num DF = numerator degrees of freedom, Den DF = denominator degrees of freedom for the corresponding sum of squares. F Value = value of the corresponding F distribution. Pr > F = p-value.

## Discussion

Assessing the efficacy of surgical device performance ex vivo is dependent upon the quality of the experimental design, and the ability of the test to control or measure those variables that contribute to device performance. Careful design of the experimental test matrix is an important first step in this process. Failure to appropriately consider practical limitations on randomization can substantially compromise the utility and significance of the data generated. The biostatistical methods described in this report provide a procedural model for the development of the design of factorial experiments that have randomization restrictions. Since the impact of more than two factors on a response variable were assessed in this experiment, it was appropriate to carry out an analysis of variance. However, an underlying assumption for the validity of the usual analysis of variance for a complete factorial design is that the runs are observed in a completely randomized order [8]. This usually requires frequent changes in the levels of the factors, which proved to be problematic in this experiment. While it was possible to randomly assign one of the two tissue types to an experimental run, there were several constraints on the other two factors due to the setup of the experiment. Solution and temperature were the hard-to-change factors. Changing the temperature required considerable time and effort, as the solution, the tissue and the apparatus needed to be adjusted to the new temperature. Changing the solution required a complete disassembly of the apparatus since the entire apparatus had to be flushed, cleaned and dried before a different solution could be employed. Due to these constraints, a completely random order of the 480 runs was not feasible. Instead, a split-plot design was used [9]. A split-plot experiment is a blocked experiment, but the blocks themselves are experimental units for a subset of the factors. The blocks are called whole plots, while the experimental units within blocks are split plots. This introduces two levels of randomization: The random order of the whole plots, and the random assignment of the treatments within the whole plots. This approach has been successfully used in industrial experiments involving factors that are hard or costly to change [7,10-13]. In the experiment described here, the whole plots consisted of the sessions (with solution and temperature fixed in each session), and each type of vessel was observed five times within each session (in random order). Using this experimental design and analysis, it was determined that the combination of temperature, solution, and vessel type affected seal quality in a statistically significant way. Therefore, experimentally, it is apparent from these data that all three of these factors need to be considered when developing an ex vivo test method for the assessment of harmonic scalpel performance and device optimization.

Acute collateral damage, although expected, was not examined in this study. Histopathology is commonly used to estimate the extent and degree of thermally-induced coagulative necrosis and protein denaturation incurred in relation to the distance from the point of energy application [14]. However, measurements of tissue injury margins obtained by histopathology may not reflect the true distance of thermal impact due to the tissue shrinkage that occurs during processing [15]. Several methods exist for determining temperature elevation and thermal spread in tissues at and at fixed distances from the energy source [16]. However, differences exist between in vivo and ex vivo analyses of collateral damage and thermal spread, especially at larger distances from the blade [17]. These differences are likely due to the effects of perfusion on the efficient removal of heat and the rapid activation of the innate acute wound response in vivo [17]. Nevertheless, the assessment of collateral tissue damage is an important aspect of surgical device performance. In future experiments, measurements of collateral damage obtained through histopathology along with temperature elevation and thermal spread can be incorporated as response variables within the optimized test-matrix described here to provide a more comprehensive analysis of device performance ex vivo.

## Conclusions

From this investigation, it was determined that the design and implementation of a split-plot experimental test-matrix provided a mechanism for addressing the existing technical randomization restrictions in a statistically meaningful way, while preserving the ability to examine the potential combinatorial effects of independent factors or variables that can be assessed in ex vivo testing. This type of approach would likely be useful to a broad spectrum of medical device researchers who must attempt to recapitulate in vivo conditions, ex vivo.

## Competing interests

This research was funded by an industrial research contract from Ethicon Endo-Surgeries, Blue Ash, OH, to the University of Dayton. Publication costs are being defrayed by the University of Dayton through an unrestricted research account (CMK). The authors do not hold any stocks or patents related to this work.

## Authors' contributions

WSD carried out the design of the experiment, generated the split plot design with randomization restriction, and performed the statistical analyses.

CMK jointly conceived of the study (with MFP), participated in the design and implementation of the experimental test set up, trained student research assistants, conducted vessel burst pressure testing, contributed to the interpretation of the data analysis, and jointly oversaw project coordination.

MFP jointly conceived of the study (with CMK), participated in the design and implementation of the experimental test set up, trained student research assistants, conducted vessel burst pressure testing, performed the data analysis, and jointly oversaw project coordination. All co-authors contributed to the preparation of the manuscript, and have read and approve the final manuscript.

## Pre-publication history

The pre-publication history for this paper can be accessed here:

http://www.biomedcentral.com/1471-2288/11/75/prepub
